# Central Pancreatectomy: Balancing between the Favorable Functional Results and the Increased Associated Morbidity

**DOI:** 10.1055/s-0044-1782655

**Published:** 2024-03-14

**Authors:** Dimitrios Symeonidis, Ismini Paraskeva, Athina A. Samara, Labrini Kissa, Alexandros Valaroutsos, Eleana Petsa, Konstantinos Tepetes

**Affiliations:** 1 Department of Surgery, University Hospital of Larissa, Mezourlo, Larissa, Greece

**Keywords:** central pancreatectomy, functional results, morbidity, postoperative pancreatic fistula

## Abstract

**Introduction** Central pancreatectomy (CP) represents an organ-preserving type of pancreatic resection. The procedure has been associated with improved long-term functional results, but increased postoperative morbidity rates, compared with the more radical resection types. The purpose of the present study was to present the outcomes of three consecutive CPs performed in our department.

**Materials and Methods** Between January 2021 and January 2022, three patients (A, B, and C) were submitted to a CP in our department. Relevant patient data including data of the detailed preoperative assessment, operations notes, and recovery charts were prospectively collected and reviewed for all subjects. A scheduled follow-up, at the outpatient clinic, was conducted to assess the long-term functional results.

**Results** The postoperative course of patient A, a 56-year-old male, was complicated by a grade C postoperative pancreatic fistula that required a reoperation. Patient B, a 66-year-old female, developed a biochemical leak that resolved spontaneously while patient C, a 64-year-old male, had a completely uneventful recovery. The length of hospital stay for the three patients was 24, 12, and 8 days, respectively. Regarding the long-term results, patient B was lost to follow-up while both patient A and C were followed up, as outpatients, 21 and 10 months after the operation. During follow-up, in patient A, we did not record the presence of symptoms consistent with pancreatic exocrine insufficiency, the hemoglobin A1C (HbA1C) levels were 7.1% while no additional medications were needed to be prescribed to maintain the glycemic control following surgery. In patient C, a significant weight loss was recorded (body mass index reduction of 11 kg/m ^2^ ) without however the presence of malabsorption-specific symptoms. The HbA1C levels were 7.7% and optimal glycemic control was achieved with oral antiglycemic agents alone.

**Conclusion** CP should be regarded as a type of pancreatic resection with certain and very limited oncological indications. An approach of balancing the advantages out of the superior postoperative functional results with the drawbacks of the increased procedure-associated morbidity could highlight the patient group that could potentially experience benefits out of this limited type of resection.

Surgery plays a key role in the management of patients with pancreatic lesions. According to the location of the lesion within the pancreatic parenchyma, a pancreaticoduodenectomy, a distal pancreatectomy (DP) or, even, a total pancreatectomy could be indicated. 1 As pancreatic cancer is a highly lethal malignancy, the oncological results of the surgical treatment, unavoidably, become the primary endpoint in the majority of the relevant assessment audits. 1 Within this framework, the functional outcomes following pancreatic surgery are not usually sufficiently appreciated. In general, the functional recovery after any form of pancreatic resection is associated with the disturbed gastric and duodenal function and with the significant changes at the level of pancreatic, endocrine and exocrine, and hormonal function. 2

The alteration of the normal anatomy, following pancreatic resections, results in significant pathophysiological changes affecting the whole process of digestion. 2 Conditions such as the delayed gastric emptying which implies the disturbed propagation of gastric contents can complicate both the immediate and late patients' recovery. In addition, the decreased levels of pancreatic stimulating hormone along with the insufficiency of the exocrine part of the pancreas can lead to defective absorption of nutrients. 3 However, as the pancreas has a dual, both endocrine and exocrine, function endocrine insufficiency, manifested mainly as diabetes mellitus, can also occur. 3 In general, these hormonal insufficiencies seem to be directly related to the extent of pancreatic parenchymal resection and usually have a profound impact on patients' quality of life. 3

Provided that the oncological indications are not compromised and aiming to decrease the impact of pancreatic resections on the quality of patients' life, tissue-preserving pancreatic resections have been proposed. 4 Patients with tumors of certain characteristics, regarding the location and the biological aggressiveness of the disease, could be candidates for these limited resection types. Thus, for tumors in the neck or proximal body of the pancreas, in particular benign and low-grade malignant lesions, tissue-preserving pancreatectomies such as pancreatic enucleation (PE) or central pancreatectomy (CP) could be employed. 4 5 PE has been proposed as a safe alternative to major resections for benign tumors such as islet cell tumors and cystic tumors with better short-term outcomes, comparable mortality but significantly better postoperative endocrine and exocrine pancreatic function compared with the more extensive resection types. 4 5 6

On the other hand, CP represents the alternative, mainly to DP, when a tissue-preserving resection could be advocated but PE is contraindicated. The most common indications include patients with neuroendocrine neoplasms, cystic tumors, metastases, and other rare benign disease located in the pancreatic body. 7 Regarding the functional outcomes, CP has been indeed associated with a significantly lower incidence of endocrine and exocrine pancreatic insufficiency, that is, 2 and 6%, respectively, compared with DP. 7 8 9 However, CP has been associated with increased morbidity and more specifically with increased incidence of postoperative pancreatic fistula (POPF) that could counterbalance the possible functional benefits. 7 10

The purpose of the present article was to present the outcomes of three consecutive CPs performed in our department. We looked primarily for the short-term results of the procedure, in terms of immediate postoperative morbidity, but the long-term functional results were also to be assessed.

## Material and Methods

Internal board approval and ethics committee permission were obtained prior to the initiation of this study. Between January 2021 and January 2022, three patients (A, B, and C) were submitted to a CP in our department. Relevant patient data including data of the detailed preoperative assessment and staging, operations notes, and postoperative recovery charts were prospectively collected and reviewed for all subjects.

Patients were evaluated in the department's multidisciplinary team meeting and a decision for surgery was made in all cases. After a thorough preoperative assessment, all patients were deemed fit for general anesthesia and surgery. All patients were submitted to CP via a bilateral subcostal incision under general anesthesia. Regarding the surgical technique, we aimed in preserving both the gastroduodenal and the splenic arteries. After dissecting free the pancreas from the superior mesenteric–portal vein and the superior mesenteric artery, the pancreatic parenchyma was transected proximally with the use of a linear stapling device taking special care in preserving the gastroduodenal artery. The proximal main pancreatic duct stump was commonly not identified; however, the whole proximal transection surface was additionally oversewn with a 3–0 polydioxanone (PDS) continuous suture. Then, the pancreatic lesion was mobilized from the splenic vein taking extra care in identifying and preserving the splenic artery. The dissection was performed distally, toward the tail of the pancreas, to achieve clear resection margins. We used a pointed scalpel to transect the body of the pancreas distally. Adequate hemostasis on the distal transection surface was achieved with the use of bipolar cautery and with properly placed figure of eight sutures avoiding the incorporation of the main pancreatic duct within the hemostatic sutures.

The distal pancreatic stump was then mobilized from the splenic vein, up to 2 cm in length, and a two-layer pancreaticogastrostomy was, then, created. More specifically, a gastrotomy was performed on the anterior gastric wall to gain access into the lumen of the stomach. Then, an additional incision on the posterior gastric wall was performed under direct vision. In general, the length of the incision was slightly smaller than the diameter of the distal pancreatic transection surface. Two anchor sutures were then placed, on the pancreatic transection surface. Pulling the two pancreatic anchor sutures through the two gastrotomies we achieved the invagination of the pancreatic stump into the gastric lumen. Then, through the anterior gastrostomy, the inner layer of the pancreaticogastrostomy was created with interrupted absorbable monofilament 3–0 PDS sutures. Usually, 6 to 8 sutures were sufficient for the completion of the inner layer. Then, the outer layer was performed, in a similar manner, with circumferentially placed interrupted monofilament 3–0 PDS sutures. Finally, two drains were left in place, that is, a left-sided drain at the region of the pancreaticogastrostomy and a right-sided drain at the region of the stapled proximal stump.

Postoperatively, patients were closely monitored for early diagnosis and treatment of procedure-associated complications. Amylase levels in the drain output were routinely assessed every 72 hours starting on postop day 3. If amylase levels were greater than 300 mg/dL, then octreotide (100 µg every 8 hours intravenously) would be administered. The administration of parenteral metoclopramide (10 mg every 8 hours intravenously) was routine following surgery. Normal diet was resumed as soon as weaning from the nasogastric tube was possible.

After discharge, a follow-up, as outpatients, was scheduled to assess the long-term functional results following the procedure. Patients were asked specific questions regarding the presence of common symptoms of pancreatic exocrine insufficiency such as weight loss, diarrhea, steatorrhea, abdominal pain, and bloating. 8 9 The possible occurrence of new-onset or aggravation of existing diabetes mellitus was assessed. In addition, aiming to objectify the endocrine pancreatic function assessment, blood samples were obtained to determine hemoglobin A1C (HbA1C) levels.

## Results

Patient A was a 56-year-old male (body mass index [BMI]: 38 kg/m ^2^ ) with a past medical history of type II diabetes mellitus with preoperative HbA1C levels of 6.7% and arterial hypertension. He was incidentally diagnosed with a mucinous cystic pancreatic neoplasm, 5.3 cm in diameter, located in the body of the pancreas. The diagnostic workup included a computed tomography (CT) scan and a magnetic resonance imaging (MRI) of the pancreas. An endoscopic ultrasound (EUS) combined with fine-needle aspiration (FNA) was performed as well, which confirmed the presence of the cystic neoplasm. Cytology did not confirm the presence of malignant cell.

Patient B was a 66-year-old female (BMI: 18 kg/m ^2^ ) with a past medical history of arterial hypertension. An imaging workup in the direction of the localization of a possible insulinoma was performed because of the presence of Whipple's triad along with high serum levels of insulin. 11 The CT scan confirmed the presence of a 2-cm diameter nodule in the body of the pancreas with imaging characteristics consistent with insulinoma. The EUS-guided biopsy of the lesion confirmed the imaging diagnosis.

Finally, patient C was a 64-year-old male (BMI: 36 kg/m ^2^ ) with a past medical history of refractory type II diabetes mellitus on insulin therapy with preoperative HbA1C levels of 9.9% and hyperlipidemia. The presence of atypical abdominal signs such as intermittent epigastric pain and bloating dictated the imaging investigation with a CT scan which revealed a cystic neoplasm of the body of the pancreas consistent with a main duct intraductal papillary mucinous neoplasm. The MRI with magnetic retrograde cholangiopancreatography confirmed the diagnosis while the diagnostic workup was complemented with EUS and FNA.

The postoperative course of patient A was complicated by a grade C POPF according to the International Study Group of Pancreatic Surgery (ISGPS) definition. 10 Starting on postoperative day 3, the left-sided drain output was 250 mL/24 hour with an amylase content of 5350 IU/L. However, on postoperative day 4, spikes of fever along with the elevated markers of inflammation dictated an imaging evaluation with CT abdomen which revealed a fluid collection with air bubbles in the anatomic area of the pancreas that was not accessible to CT-guided drainage. A decision for an urgent laparotomy was made. During laparotomy, the collection was drained and copious irrigation of the peripancreatic area, with several liters of normal saline, was conducted. The initially placed drains, upon the index operation, were repositioned accordingly. Clinical improvement was then prompt while a POPF of low output was subsequently established. However, the occurrence of delayed gastric emptying further prolonged the length of hospital stay. Oral feeding was recommenced on postoperative day 21 and the patient was discharged on postoperative day 24.

Patient B developed a biochemical leak (low right drain output of fluid with an amylase content of 1,800 IU/L) of minor clinical importance. Oral feeding was recommenced uneventfully, and the drains were removed on postoperative day 8. The patient was discarded on postoperative day 12. Finally, patient C had an uneventful recovery. Oral feeding was recommenced on the 5th postoperative day and the patient was discharged on postoperative day 8. The pathology reports confirmed the preoperative working diagnoses in all three cases. Although a proper lymph node dissection was not a goal in this operative approach, the lymph nodes retrieved and identified by the involved pathologists were 9, 3, and 2 lymph nodes in patients A, B, and C, respectively. No metastatic lymph node involvement was documented in any of the examined lymph nodes.

Following discharge, all three patients were assigned to our department's aftercare plan designed for patients undergoing pancreatic resections which includes regular visits to the outpatient clinic for endocrine and nutritional consultation and guidance. Regarding the long-term follow-up, patient B was lost to follow-up while both patient A and C were followed up 21 and 10 months after the operation. In patient A, we did not record any symptoms consistent with pancreatic exocrine insufficiency such as weight loss, diarrhea, steatorrhea, and abdominal pain or bloating. In addition, the HbA1C levels, on follow-up, were 7.1% while no additional medications were needed to be prescribed to maintain the glycemic control following surgery. In patient C, surprisingly a significant improvement on the glycemic control was noted approximately 3 months following the procedure. The HbA1C levels were decreased to 7.7% while insulin was no longer required and optimal glycemic control was achieved with oral antiglycemic agents alone. However, a significant weight loss was recorded, that is, a BMI reduction of 11 kg/m ^2^ . Decreased appetite leading to decreased caloric intake was identified as the major cause of the weight loss. Symptoms consistent with malabsorption secondary to a possible pancreatic exocrine insufficiency were absent ( Table 1 ).

**Table 1:** Patients included in the study

Patient	Age	Past medical history	Indication	POPF grade	Length of hospital stay (d)	Pancreatic exocrine insufficiency	New-onset or aggravation of existing DM
A	56	DM type II hyperlipidemia	Mucinous cystic neoplasm	C	24	No	No
B	66	Arterial hypertension	Insulinoma	Biochemical leak	12	−	−
C	64	DM type II	Main duct IPMN	−	8	No	No

Abbreviations: DM, diabetes mellitus; IPMN, intraductal papillary mucinous neoplasm; POPF, postoperative pancreatic fistula.

## Discussion

In practice, the ideal pancreatic resection is a resection that fulfills the following three criteria: (1) is aligned with the fundamental oncological principles, (2) preserves the exocrine and endocrine pancreatic function, and (3) is associated with acceptable morbidity and mortality rates. Traditionally, testing the validity of CP in each of these three categories has been the actual challenge in the literature. Regarding the oncological appropriateness of the procedure, careful patient selection is of paramount importance. Certainly, patients with high malignant pancreatic adenocarcinoma are not candidates for the procedure. However, a thorough preoperative evaluation with detailed imaging assessment could discriminate those patients with either benign or low-grade malignant pancreatic tumors, mainly neuroendocrine or cystic neoplasms, that would be eligible for a limited resection type such as CP.

The preservation of the pancreatic function after surgery was the main argument for the introduction of tissue-preserving pancreatic resections. The logical hypothesis that less parenchymal resection would be associated with more functional adequacy of the organ in the postoperative period seems to be confirmed by literature reports. 7 8 9 Indeed, studies report a significantly lower rate of endocrine and exocrine pancreatic insufficiency in patients submitted to CP compared with DP. 7 8 9 However, the increased reported morbidity, mainly in the form of POPF development, represents the major drawback of the approach. 9 A usually encountered problem during the evaluation of the results of different studies assessing the safety and efficiency of CP was, at least initially, the lack of a universally adopted definition of pancreatic fistula, that is, the most dramatic complication following any form of pancreatic resection. In 2005, the ISGPS, aiming to overcome these problems, developed a definition and grading system for POPF which was further updated in 2016 by incorporating all the emerging literature data on the subject. 10 12 In the updated grading scheme, the previous grade A POPF is now called a “biochemical leak” while clinically significant POPF fall into the grade B or C categories, depending on their impact on the patient's clinical course.

In the present report, we present the cases of three patients submitted to CP in our department. Provided that the oncological appropriateness of the procedure was ensured by the favorable oncological characteristics of the tumors in all three patients, we chose this limited type of resection because we hypothetically aimed for the best possible long-term functional results. Having in mind the certain medical background of each patient, the theoretical advantages out of a tissue-preserving resection could possibly become even more appreciated. For example, patient A was an extremely obese 56-year-old male with ill-controlled type II diabetes due to poor compliance to the prescribed medication. Patient B was an underweight 66-year-old female in whom the possible establishment of postoperative pancreatic exocrine insufficiency would further aggravate the patients already deranged nutritional status. Finally, patient C was a 64-year-old extremely obese male with a past medical history of refractory type II diabetes mellitus. In both patient A and C, a type of resection that could be associated with a minimal impact on the endocrine pancreatic function was the actual challenge.

Because of the association of the procedure with the increased rate of POPF, we chose to secure the closure of the proximal pancreatic stump using both staplers and sutures while we anastomosed the distal pancreatic stump with the stomach. Choosing pancreaticogastrostomy over pancreaticojejunostomy was based on the postulated lower rate of POPF following pancreaticogastrostomy compared with pancreaticojejunostomy, especially in the high risk for POPF patients. 9 However, there are indeed reports that neglect the role of the type of reconstruction on the incidence of POPF following CP. 9 Among the three patients included in the present report, one patient developed a clinically significant grade C POPF, and one patient developed a biochemical leak. 12

Regarding the long-term results, we managed to get in touch and follow-up only two of the three patients included in the study. Patient B was lost to follow-up. In general, the evaluation of the exocrine pancreatic function can be quite problematic even when sophisticated pancreatic function tests such as the pancreolauryl test or the stool elastase test are utilized. 13 Only profound and usually clinically significant pancreatic insufficiency can be documented with the use of these tests. 13 Having in mind the innate limitations of the laboratory pancreatic exocrine function tests, we aimed to document the presence of possible pancreatic exocrine insufficiency in the postoperative setting by trying to elicit symptoms of relatively high specificity for exocrine insufficiency such as diarrhea and steatorrhea. Patient A did not report the presence of any symptom that could be attributable to pancreatic exocrine insufficiency. In addition, the HbA1C levels, during follow-up, were not significantly altered and the preoperative treatment plan was adequate in achieving glycemic control in the postoperative setting as well. Regarding patient C, we documented a significant weight loss without however any other symptoms consistent with pancreatic exocrine insufficiency. The HbA1C levels were notably decreased, that is, from 9.9% preoperatively to 7.7% during follow-up and insulin was no longer needed to supplement oral antidiabetics to maintain blood glucose levels within the normal range. The beneficial effect of weight loss on plasma glucose levels could possibly explain this favorable outcome.

Certainly, our goal was not to test the efficiency of CP as a valid type of pancreatic resection. The small number of patients included in the study precludes any solid conclusions regarding the effect of the technique on the recorded outcomes. However, provided that the hypothesis that associated CP with more favorable functional long-term results compared with the more radical resection types is true, this report underlines the fact that there are indeed patients that, at least in theory, could appear as ideal recipients. Well-designed studies, optimally with a long follow-up, are needed to evaluate the true role of this organ-preserving approach in the treatment of patients in whom the preservation of pancreatic function could outperform the increased, associated with the procedure, morbidity.

In conclusion, CP should be regarded as a type of pancreatic resection with certain and very limited oncological indications. Carefully balancing the advantages out of the superior postoperative functional results and the drawbacks of the increased associated morbidity would highlight the patient group that could potentially experience benefits out of this organ-preserving approach.
